# Effect of Soybean Oil and Fish Oil on Lipid-Related Transcripts in Subcutaneous Adipose Tissue of Dairy Cows

**DOI:** 10.3390/ani10010054

**Published:** 2019-12-26

**Authors:** Einar Vargas-Bello-Pérez, Massimo Bionaz, Macarena Garrido-Sartore, Nathaly Cancino-Padilla, María Sol Morales, Jaime Romero, Heidi Leskinen, Philip C. Garnsworthy, Juan J. Loor

**Affiliations:** 1 Departamento de Ciencias Animales, Facultad de Agronomía e Ingeniería Forestal, Pontificia Universidad Católica de Chile, Casilla 306, Santiago 6904411, Chile; mgarrido1@uc.cl (M.G.-S.); nlcancino@uc.cl (N.C.-P.); 2 Department of Veterinary and Animal Sciences, Faculty of Health and Medical Sciences, University of Copenhagen, Grønnegårdsvej 3, DK-1870 Frederiksberg C, Denmark; 3 Department of Animal and Rangeland Sciences, Oregon State University, Corvallis, OR 97331, USA; 4 Departamento de Fomento de la Producción Animal, Facultad de Ciencias Veterinarias y Pecuarias. Universidad de Chile. Av. Santa Rosa, La Pintana, Santiago 11735, Chile; smorales@uchile.cl; 5 Laboratorio de Biotecnología en Alimentos, Unidad de Alimentos, Instituto de Nutrición y Tecnología de los Alimentos, Universidad de Chile, Avda. El Libano 5524, Macul, Santiago 7830490, Chile; jromero@inta.uchile.cl; 6 Milk Production, Production Systems, Natural Resources Institute Finland (Luke), FI-31600 Jokioinen, Finland; heidi.leskinen@luke.fi; 7 School of Biosciences, Sutton Bonington Campus, The University of Nottingham, Loughborough LE12 5RD, UK; Phil.Garnsworthy@nottingham.ac.uk; 8 Mammalian NutriPhysioGenomics, Department of Animal Sciences and Division of Nutritional Sciences, University of Illinois, Urbana, IL 61801, USA; jloor@illinois.edu

**Keywords:** fat supplementation, transcriptomic, subcutaneous adipose tissue, lactating cows

## Abstract

**Simple Summary:**

The objective of this study was to determine the effects of degree of unsaturation of dietary lipids on lipid-related metabolites and transcription of genes involved in lipid metabolism in subcutaneous adipose tissue (SAT) of mid-lactating dairy cows. The objective was achieved by supplementing dairy cows with soybean oil (high in linoleic acid) or fish oil (high in polyunsaturated fatty acids) for 63 days (nine weeks). Results revealed effects of oil supplement on lipid metabolism but a mild effect on the transcriptome of the adipose tissue. Compared to soybean oil, fish oil had a higher lipogenic effect in SAT.

**Abstract:**

The objective of this study was to determine the effect of long-term supplementation of unsaturated oil on lipid metabolism and transcription of genes involved in lipid metabolism in subcutaneous adipose tissue (SAT) of mid-lactating dairy cows. The objective was achieved by supplementing dairy cows with soybean oil (SO; high in linoleic acid) or fish oil (FO; high in EPA and DHA) for 63 days (nine weeks). Cows were fed a control diet with no added lipid, or diets containing SO or FO (n = 5 cows/group). At the onset of the experiment (day 0) and on days 21, 42, and 63 of supplementation, blood and SAT samples were collected from each animal. Oil supplementation increased cholesterol and NEFA in plasma, with a greater effect of SO compared to FO. Concentration of BUN was lower in SO compared to control and FO at the end of the trial. Transcription of few genes was affected by dietary lipids: *FABP4* had lowest expression in FO followed by SO and control. *ACACA* and *FASN* had higher expression in FO. Transcription of *SCAP* was higher but expression of *INSIG1* was lower in SO. Overall, results revealed that compared to control, SO and FO had lipogenic effect in SAT.

## 1. Introduction

Different oils from vegetable and/or marine sources have been used to modulate milk fatty acids (FA) profile toward a healthier dairy product for human health [1]. Addition of soybean oil in dairy cow diets is effective in decreasing saturated FA (SFA) and increasing monounsaturated (MUFA) content in milk [2], while fish oil supplementation increases contents of some polyunsaturated FA (PUFA) such as eicosapentanoic acid (EPA; C20:5n-3) and docosahexaenoic acid (DHA; C22:6n-3) in milk [3].

The FA profile of the different dietary oils used in our previous experiments, such as soybean oil [2], fish oil [3], and/or hydrogenated vegetable oil [2,3], varied widely: from a saturated FA profile (hydrogenated vegetable oil was mostly from palmitic and stearic acids) to polyunsaturated FA profiles (soybean oil was more than 50% linoleic acid, whereas fish oil was 20% EPA and 17% DHA). These FA profiles elicited different effects on milk FA composition. The effect on milk fat composition is partly due to the dietary FA composition but can be also driven by the effect on the transcription of genes related to lipid metabolism in mammary and subcutaneous adipose tissues [4]. However, these molecular effects remain largely unexplored. On the other hand, the number of double bonds and location of double bonds of dietary oils result in different biohydrogenation intermediates due to the activity of the rumen. Those intermediates can affect the lipid metabolism differently [5], partly through effects on transcription of lipogenic genes. Dietary long-chain fatty acids (LCFA) are known to exert evident nutrigenomic effects at cellular [6] and subcutaneous adipose tissue (SAT) [7] levels in dairy cows. 

After the mammary gland, SAT is one of the most important tissues concerning lipogenesis [8]. Unfortunately, most published studies that focused on nutrigenomic effects on SAT of supplemented dietary LCFA in dairy cows only lasted up to four weeks [9,10], which is a relatively short period considering the life cycle of dairy cows. Long-term studies need to be conducted to assess the long-term nutrigenomic consequences of lipid supplementation on SAT. Recently, we observed a differential long-term nutrigenomic response in SAT of dairy cows between dietary supplementation of olive oil (unsaturated LCFA) or completely hydrogenated vegetable oil [7]. Prior in vitro studies indicated that the transcriptomic effect of LCFA in bovine epithelial cells is proportional to the degree of saturation [11]. Those data indicate that the level of saturation of LCFA is an important determinant for the nutrigenomic property of the dietary LCFA in bovine; however, the effect of the number of double bonds of dietary LCFA on the transcriptome of SAT in bovine is not known.

The objective of this study was to determine the effect of long-term (nine weeks) supplementation of unsaturated LCFA with different number of double bonds on lipid metabolism and the transcription of genes involved in lipid metabolism in the SAT in mid-lactating dairy cows. The objective was achieved by supplementing dairy cows with soybean oil (high in linoleic acid, two double bounds) or fish oil (high in EPA—five double bounds—and DHA—six double bounds) for 63 days. The main hypothesis tested in this study was that the long-term (63 days) supplementation of two oil rich in PUFA has a differential effect on lipid metabolism and on the transcription of lipid-related genes in SAT of mid-lactating dairy cows. In particular, we expect that the number of double bonds of dietary LCFA can differentially affect the mRNA abundance of genes involved in lipid metabolism. 

## 2. Materials and Methods 

### 2.1. Animals, Experimental Diets, and Tissue Sampling

Animal care and procedures were carried out according to the guidelines of the animal care committee of the Pontificia Universidad Católica de Chile (project code 160809002). The study was conducted at the Estación Experimental Pirque of the Pontificia Universidad Católica de Chile. Cows were housed in a free-stall barn and fed using Calan Broadbent feeding doors (American Calan, Inc., Northwood, NH, USA) and had continuous access to water.

Details of the study can be found in our companion paper [12]. Briefly, 15 Holstein cows, averaging 198 ± 35 days in milk and a live weight of 637 ± 68 kg at the beginning of the study, were assigned to three treatment groups: Control, did not receive lipid supplementation; SO, supplemented with unrefined soybean oil at 2.9% DM; and FO, supplemented with unrefined salmon oil at 2.9% DM (Table 1). Five cows per group were fed on each diet for a period of nine weeks. Oils were mixed manually into the daily ration for each cow.

### 2.2. Plasma Samples and Fatty Acid Analysis

At the beginning of the study (day 0) and on days 21, 42, and 63, blood samples (50 mL/cow) were obtained at 10:00 (2 h after feeding) via jugular puncture. Blood was transferred to tubes containing lithium heparin (BD Vacutainer; Franklin Lakes, NJ, USA) and immediately centrifuged for 15 min at 3000× *g* (C-28A; BOECO, Hamburg, Germany) for harvesting plasma. Samples were stored at −80 °C, until analyzed for blood metabolites. Samples were analyzed for 3-hydroxybutyrate (BHBA), cholesterol, blood urea nitrogen (BUN), glucose, and non-esterified FA (NEFA), using commercial kits in an autoanalyzer at a certified laboratory (Laboratorio Clínico Veterinario, Universidad Austral de Chile).

### 2.3. Biopsies, RNA Extraction, and Reverse Transcription Quantitative Polymerase Chain Reaction (RTqPCR)

At the onset of the experiment (day 0) and on days 21, 42, and 63 of supplementation, SAT samples were collected from the tail-head of each cow. Details of the biopsies, RNA extraction, and RTqPCR procedures can be found elsewhere [6]. Briefly, RNA was extracted by using QIAzol Lysis Reagent (Qiagen Inc., Valencia, CA, USA) according to the manufacturer’s protocols. Genes tested in the current study and their biological functions are listed in Table 2. Primer pairs were previously reported [13], and the final PCR data were calculated by using LinRegPCR 12.18 software [14].

### 2.4. Statistical Analysis

For RTqPCR data, *GAPDH* (glyceraldehyde 3-phosphate dehydrogenase), *EIF3K* (eukaryotic translation initiation factor 3 subunit K) and *UXT* (ubiquitously expressed prefoldin like chaperone) were tested as reference genes by the geNorm algorithm [15]. All three genes had an M-value of ≤0.80, and the use of the geometrical mean of the three reference genes provided a V-value of 0.245, indicating a good normalization factor.

## 3. Results 

### 3.1. Blood Metabolites

**Figure 1:**
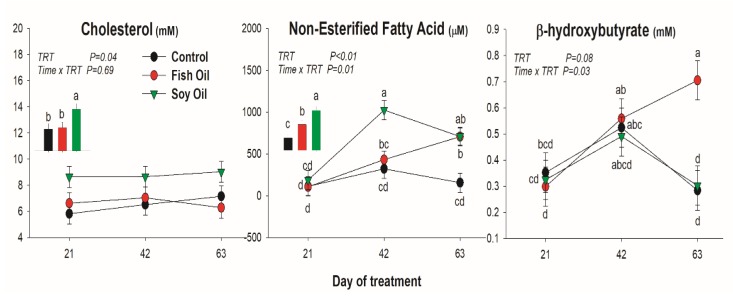
Effect on lipid-related blood metabolites of long-term supplementation of lactating dairy cows (n = 5/group) with soybean oil or fish oil. The *p*-value of the overall treatment (TRT) and TRT × Time interaction is reported. Data were corrected for the baseline before statistical analysis. Bars denote standard error of the means. Insert bar graph denote the means between treatments.

**Figure 2:**
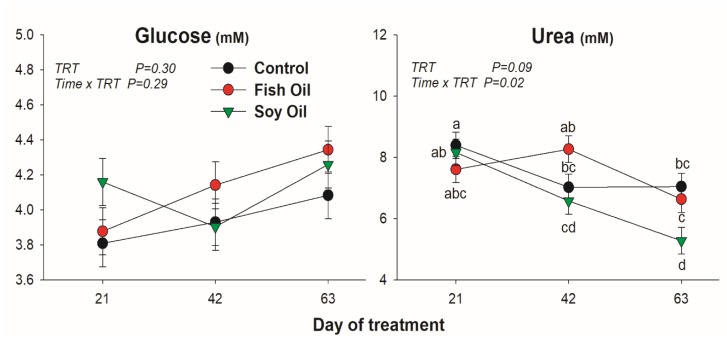
Effect on plasmatic concentration of glucose and urea by long-term supplementation of lactating dairy cows (n = 5/group) with soybean oil or fish oil. The *p*-value of the overall treatment (TRT) and TRT × Time interaction is reported. Data were corrected for the baseline before statistical analysis. Bars denote standard error of the means.

All lipid-related metabolites were affected by the treatment (Figure 1). Cholesterol was increased only by soybean oil treatment, while NEFA was increased by both oil supplementations, but with a quicker and larger increase when soybean oil was used compared to fish oil. The BHBA was only increased by fish oil after 63 days of treatment. Concentration of BUN was reduced by SO compared to the other treatments, especially at 63 day of treatment (Figure 2). Glucose was not affected by the oil supplementation (Figure 2).

### 3.2. Transcription

A summary of the changes in mRNA of genes related to lipid metabolism in SAT is shown in Table 3.

#### 3.2.1. Transcripts Related to Fatty Acid Transport and Activation

**Figure 3:**
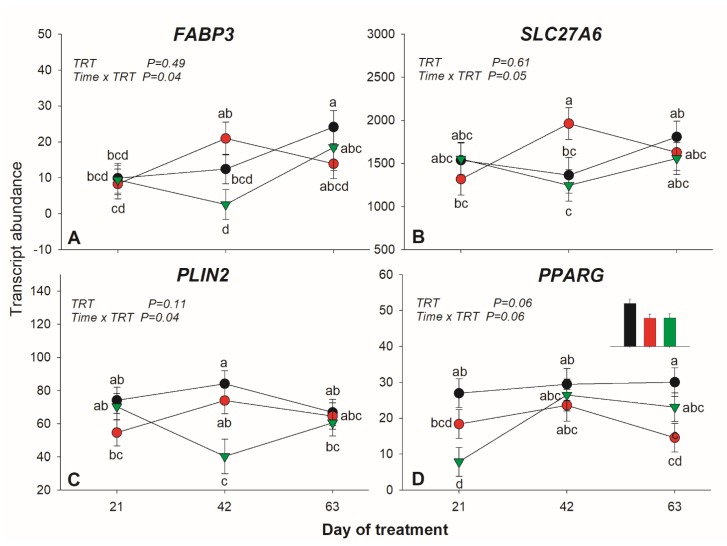
Transcription patter of genes affected by treatment × time interaction in adipose tissue of lactating dairy cows (n = 5/group) receiving a long-term supplementation of soybean oil or fish oil. *p*-value of the overall treatment (TRT) and TRT × Time interaction is reported. **A** = F*ABP3*, **B** = *SLC27A6*, **C** = *PLIN2* and **D** = *PPARG*. Data were corrected for the baseline before statistical analysis. Bars denote standard error of the means.

*ACSL1* had lower expression in SO compared to control. A tendency (*p* = 0.06) for a lower expression for *VLDLR* in SO was detected. The expression of *FABP3* and *SLC27A6* was affected by diet × time interaction with higher expression in FO vs. SO at 42 day of treatment (Figure 3A,B).

#### 3.2.2. De Novo Synthesis and Fatty Acid Desaturation

Among all the transcripts measured in this category, only *ACACA* had higher expression in FO compared to the other groups.

#### 3.2.3. Triacylglycerol Synthesis and Lipid Droplet Formation

Transcription of *PLIN2,* coding for the adipose differentiation-related protein adipophilin, was significantly reduced by SO at 42 day of treatment (Figure 3C). Of the other transcripts measured in this category, only a tendency (*p* = 0.09) for a full interaction for *DGAT1* was detected, due to a decrease in expression in SO.

#### 3.2.4. Transcription Regulation

*INSIG1* was the only affected transcript in this category, with higher expression in SO vs. FO. Expression of *PPARG* tended (*p =* 0.06) to be lower in both oil supplements compared to the control. A tendency (*p* = 0.06) for a full interaction was also observed for this transcript, due to a decrease in SO at 21 day of treatment, with a quick increase afterward (Figure 3D). No other mRNAs related to transcription regulation were affected by the treatments.

## 4. Discussion

### 4.1. Dairy Cow’s Performance and Milk Traits

The use of supplemental fats is a nutritional strategy that helps to increase the energy density of dairy cows’ diets and thereby sustains or increases milk yields [2,3]. The dietary intervention (supplementation of 2.9% DM of dietary SO or FO) chosen for this study was sufficient to provoke less contents of total SFA without deleterious effects on overall animal´s production performance. In terms of milk FA profile, SFA in milk fat were decreased with SO and FO compared with control. C18:2 cis-9, cis-12 was increased with SO, whereas C18:2 cis-9, trans-11, C20:3n-3, C20:3n-6, C20:5n-3, and C22:6n-3 were the highest with FO [12]. Overall, the observed milk FA profiles obtained from dietary SO and FO partly explain that there was an altered rumen lipid metabolism, leading to the formation of FA biohydrogenation intermediates, but these changes were not enough to provoke milk fat depression [17]. Possibly, because we used less than 5% DM of lipid supplementation and high-forage basal diet (63:37 forage to concentrate ratio), that may have resulted in only minor shifts in ruminal production of acetate and butyrate and mammary uptake of those metabolites.

### 4.2. Blood Metabolites

Measuring plasma biomarkers when cows are fed with supplemental fat during mid-lactation is important to have some indicators of lipid metabolism and energy balance. Blood metabolites varied over the time and those changes were expected due to advancing lactation stage. Cholesterol, as index of lipoproteins production, and NEFA are expected to increase in plasma due to fat supplementation in dairy cows [18,19]. NEFA in plasma derived by the lipolysis of the SAT during negative energy balance which is typical of early postpartum animals; however, cows in our experiment were in mid-lactation and were not expected to be in negative energy balance. Thus, NEFA increase in plasma in our experiment was driven by the lipoprotein activity on triglycerides-rich intestinal-derived VLDL. Interestingly, only SO increased cholesterol in our study, which is an index of lipoproteins. A triglyceride-lowering effect of omega 3 FA are well-known in humans by several mechanisms, including the increased utilization of fatty acids by peripheral tissues, increasing LPL activity, and decreasing inflammation [20]. Interestingly, the same treatment in our study increased the level of plasmatic BHBA. However, with the existing data, it is unclear what the mechanism is that is driving this effect. Certainly, the FO treatment in our study did not affect the uptake of triglycerides from lipoproteins via lipoprotein lipase, as previously reviewed [20], since the transcription of *LPL* was not affected in SAT by treatments in our study. 

Blood urea concentration is influenced by a wide variety of interrelated parameters including dietary protein intake and protein requirements for milk production and protein metabolism [21]. Normally, the main cause of high circulating urea is an excess intake of total N, including rumen degradable protein [22]. Another reason for increased urea production is an energy deficit, which stimulates catabolism of amino acids from tissue proteins, leading to increased urea production [21]. It is unclear in our study the reason for the lower urea in SO. 

### 4.3. Effects of Dietary SO and FO on Lipid-Related Genes in Subcutaneous Adipose Tissue

Contrary to our hypothesis, we only detected few transcripts in the SAT that were affected by long-term supplementation of SO and FO. The higher transcription of *ACACA* and lower *INSIG1* in FO compared to the other groups indicated a larger de novo synthesis [13]. Overall, data indicated a tendency for a decreased import and activation of long chain FA in SAT, especially in SO, as suggested by the decrease in the transcription of *FABP3*, *SLC27A6,* and *ACSL1* in SO. The decreased import of LCFA might also explain the highest peak of NEFA observed in this group. It is possible that the high NEFA in SO have also increased the accumulation of triglycerides in the liver. This could have compromised the urea cycle [23]. 

The SREBP1 plays a fundamental role in cholesterol and FA metabolism [24]. The change in expression of *INSIG1*, assuming a proportional translation, would likely affect the activity of *SREBP1*, one of the main regulators of de novo FA synthesis in the adipose tissue [25]. *INSIG1* works as an inhibitor of SREBP1 activity [13]. The two oil supplements had a reverse effect on the expression of *INSIG1*, with FO decreasing its expression and SO increasing it. This change appeared to have partly affected the expression of *ACACA*, a key de novo FA synthesis gene and a putative SREBP1 target gene. The concomitant decrease in expression of LCFA transporters and activation (i.e., *FABP3*, *SLC27A6*, and *ACSL1*) support a decrease of lipogenesis in SAT of SO treated cows. The decreased lipogenesis is also supported by a decrease in expression of *PPARG* during the early phase of supplementation. The FO treatment appeared to have increased de novo FA synthesis. Increase in de novo fatty acid synthesis in SAT was also previously observed in dairy cows infused with CLA for 4 days [8]. The effect in the transcriptome of that study was larger than what observed in our study and, for some of the transcripts the effect was the opposite, such as the higher expression of *PPARG* in the study of Harvatine et al. [8] while it was downregulated in our study. This is consistent with the fact that we did not observe any milk fat depression [2,3]. The latter is also caused by a substantial decrease in expression of lipogenic genes in the mammary tissue and an increase in transcription of similar genes in the adipose tissue [8]. The original purpose of the study was to increase the amount of dietary unsaturated LCFA without inducing milk fat depression, which we achieved successfully [2,3]. Thus, the lack of large change in transcription of measured lipogenic gene might also be due to the amount and/or types of oil used. 

Our data indicated an opposite effect on lipogenesis in SAT by the two PUFA supplementation. However, only few transcripts were affected by the treatments. Thering et al. [9] also reported a relatively minor effect on lipogenic genes, most of them overlapping with our measured transcripts. In that work it was observed a greater expression of *LPL* and *SCD1* in SAT of cows fed a blend of SO and FO for 21 d. Both transcripts were unaffected in our study. It is unclear the reason for the observed differences; however, we fed FO and SO rather than their blend, but the doses were not extremely different. Thering et al. [9] fed 1.5% FO + 2.5% SO (i.e., 3.5% DM total supplement), while we fed 2.9% DM supplement. It is possible that the combination of the two oils provided a profile of FA that interacted with the various transcription factors in a different manner than when only one of the oils is present. Each LCFA appeared to have a differential interaction with transcription factors, as previously observed in an in vitro study using goat mammary cells [6].

Despite the fact that SO and FO can be considered as PUFA sources, the number and location of double bonds seems to exert some effects on the transcription of isogenic genes. In this study, SO had 50 g/100 g of C18:2 cis-9, cis-12, whereas FO was composed (g/100 g) mostly by C18:2 cis-9, cis-12 (16), C20:5n-3 (16), C20:5n-3 (5), and C22:6n-3 (8). That, by some means, shows the role of the chemical configuration of dietary lipids on the transcription of lipogenic genes in SAT. 

### 4.4. Limitations

Some factors need to be considered when interpreting the transcriptomic data from this study. The lack of strong effects on gene transcription in SAT may be related to the low number of animals used for each treatment. Data on size and distribution of adipocytes would have helped in determining if treatments affected adipogenesis and/or lipogenesis. The FA profile from SAT would have helped to explain the effects of treatments in transcription of genes related to de novo synthesis (e.g., *ACACA*) and import of preformed fatty acids. The quantity of supplemental fat used in this study was enough to change milk FA profile but not enough to provoke bigger transcriptomic changes in SAT. Modest nutrigenomic effects were also previously observed in mid-lactating ewes [4] and mid-lactating dairy cows [7] in positive energy balance fed with mild amounts of supplemental SO (2.5% DM) and olive oil and hydrogenated vegetable oil (2.9% DM), respectively. Another reflection from the obtained results is that rumen microbiome analysis will be highly suggested as that could help to have a clearer picture of the biological processes that took place at rumen level. It remains unknown how rumen microorganisms couple with dietary fats in a relative long-term supplementation.

Interpretations of the data in our study is limited by the absence of functional data besides the few blood metabolic markers assessed. Our interpretation of the data is also limited by the inference that change in abundance in a transcript would translate in changing of the amount of the coded protein. Although lipogenesis is highly regulated via transcriptomic changes, several lipogenic enzyme are regulated allosterically, as it has been well established for ACACA [26]. Furthermore, several parameters, such as body condition score, ultrasonography of adipose tissue depots, or adipocyte size could have helped to demonstrate a functional change in the lipogenesis rate; unfortunately, those data were not available.

## 5. Conclusions

Overall, the results indicate that oil supplements affect the lipid metabolism and the production of urea, although the mechanism for the latter remains unclear. The long-term oil supplementation had a modest nutrigenomic effect with a possible increase in de novo FA synthesis and decrease import of LCFA by FO. Minor differences were observed between FO and SO, with SO having a lower nutrigenomic effect on transcription of genes related to lipogenesis in SAT compared to FO. The latter data marginally supports the hypothesis that the number of double bonds of dietary lipids affect the mRNA abundance of lipid-related genes.

## Figures and Tables

**Table 1:** Ingredients of control, soybean oil (SO), and fish oil (FO) dietary treatments.

Ingredient Composition (% DM)	Diet		
	Control	SO	FO
Corn silage	32.0	31.1	31.1
Fresh alfalfa	24.0	23.3	23.3
Malt distillers	19.2	18.6	18.6
Corn grain	7.6	7.4	7.4
Canola meal	6.2	6.0	6.0
Alfalfa hay	5.0	4.9	4.9
Soybean grain	4.0	3.9	3.9
Wheat bran	1.6	1.6	1.6
Vitamin and mineral premix ^1^	0.4	0.4	0.4
Soybean oil	0	2.9	0
Fish oil	0	0	2.9

^1^ Contained per kg: 25 g of P; 80 g of Ca; 25 g of Mg; 1.6 g of S; 300,000 IU of vitamin A; 50,000 IU of vitamin D_3_, and 1600 IU of vitamin E.

**Table 2:** Gene symbol, name, and lipogenesis-related functions of the 20 genes evaluated in the present study.

Symbol	Name	Function
*ACACA*	Acetyl-CoA carboxylase alfa	Catalyzes the rate-limiting reaction in the de novo synthesis of LCFA
*ACSL1*	Acyl-CoA Synthetase Long Chain Family Member 1	Convert LCFA into acyl-CoA esters, transport of exogenous FA
*ACSS2*	Acyl-CoA Synthetase Short Chain Family Member 2	The chemical reactions and pathways resulting in the formation of acetyl-CoA from acetate
*PLIN2*	Adipose Differentiation-Related Protein	Involved in formation and maintenance of lipid droplets
*DGAT1* and *2*	Diacylglycerol O-acyltransferase Homolog 1 and 2	Acyltransferase that catalyzes the terminal and only committed step in triacylglycerol synthesis
*FABP3* and *4*	Fatty Acid Binding Protein 3 and 4	Intracellular transport of acyl-CoA; regulation of gene expression by providing LCFA to PPARγ
*FADS2*	Fatty acid desaturase 2	Desaturase introducing a cis double bond at carbon 6 of the fatty acyl chain
*FASN*	Fatty acid synthase	Fatty acid synthetase catalyzes the formation of long-chain fatty acids from acetyl-CoA, malonyl-CoA and NADPH
*SLC27A6*	Soluble Carrier Protein 27A	LCFA translocation (high uptake); Convert LCFA into acyl-CoA esters
*INSIG1*	Insulin Induced Gene 1	Mediates feedback control of cholesterol synthesis by controlling SCAP and HMGCR
*LPIN1*	Lipin 1	Dephosphorylation of phosphatidate yielding diacylglycerol; Transcription (PPARα co- factor)
*LPL*	Lipoprotein Lipase	Catalyzes the hydrolysis of triglycerides from circulating chylomicrons and very low-density lipoproteins
*PPARG*	Peroxisome Proliferator Activated Receptor Gamma	Regulate transcription of lipogenic and adipogenic genes.
*SCAP*	SREBP Chaperone	Protein required for cholesterol as well as lipid homeostasis. Chaperone for activation of SREBP1
*SCD1*	Stearoyl-CoA desaturase 1	Desaturase introducing introduce the first double bond into saturated fatty acyl-CoA substrates
*SREBP1*	Sterol Regulatory Element Binding Transcription Factor	Transcriptional regulation of cholesterol synthesis and lipogenesis genes
*THRSP*	Thyroid Hormone Responsive	Nuclear protein which is important in the regulation of lipid metabolism
*VLDLR*	Very Low-Density Lipoprotein Receptor	Binds very low-density lipoproteins assisting LPL

Outliers were checked, using Proc Reg of SAS (v.9.4, SAS Institute Inc., Cary, NC, USA), removing data with a studentized t > 3.0. Data were arithmetically adjusted at time 0 between groups, as previously described [16], and time 0 was removed from the statistical model. Data were analyzed by using the MIXED procedure in SAS (SAS Institute Inc., Cary, NC, USA), using diet (Control, FO, and SO), time (21, 42, and 63 day of treatment), and diet × time as fixed effects and cow within diet as random effect. Least squares mean (LSM) were separated using the PDIFF statement in SAS. A *p*-value ≤ 0.05 was declared as significant and *p*-value ≤ 0.10 as tendency.

**Table 3:** Transcript abundance of genes related to lipid metabolism in subcutaneous adipose tissue (SAT) of cows supplemented with soybean oil (SO), and fish oil (FO).

Gene	Treatment			SEM	*p*-Value		
	Control	FO	SO		Diet (D)	Time (T)	D × T
Fatty acid transport and activation							
*ACSL1*	77.0 ^a^	68.9 ^ab^	52.5 ^b^	6.72	0.04	0.39	0.60
*FABP3*	15.5	14.4	10.1	3.40	0.49	0.03	0.04
*FABP4*	525.9	379.6	477.9	100.7	0.59	0.04	0.31
*LPL*	131.5	138.6	154.4	30.4	0.86	0.98	0.90
*SLC27A6*	1569.4	1635.7	1451.4	134.7	0.61	0.37	0.05
*VLDLR*	6.20	6.74	5.16	0.43	0.06	0.13	0.24
De novo synthesis and fatty acid desaturation							
*ACACA*	27.9 ^b^	39.1 ^a^	20.3 ^b^	3.95	<0.01	0.58	0.82
*ACSS2*	53.1	67.3	67.2	19.8	0.85	0.07	0.53
*FADS2*	2.40	1.77	1.87	0.43	0.54	<0.01	0.56
*FASN*	33.4	129.2	106.9	36.6	0.18	0.31	0.90
*SCD1*	356.6	545.9	500.8	160.7	0.69	0.16	0.75
Triacylglycerol synthesis and lipid droplet formation							
*DGAT1*	33.3	32.3	25.0	2.98	0.11	<0.01	0.09
*DGAT2*	122.5	167.0	102.7	46.6	0.58	0.06	0.54
*LPIN1*	8.81	6.89	8.25	1.90	0.76	0.22	0.26
*PLIN2*	75.0	64.4	57.0	5.81	0.11	0.91	0.04
Transcription regulation							
*INSIG1*	2.07 ^ab^	1.71 ^b^	2.79 ^a^	0.28	0.04	<0.01	0.96
*PPARG*	28.8	18.8	19.1	2.97	0.06	0.02	0.06
*SCAP*	6.90	5.57	8.17	0.81	0.12	<0.01	0.63
*SREBP1*	26.0	30.1	28.5	2.29	0.44	0.09	0.36
*THRSP*	37.8	85.6	78.9	25.1	0.45	0.37	0.66

^a,b^ values within the same row indicate the significant differences among treatments.
